# Important prognostic factors for the long-term survival of lung cancer subjects in Taiwan

**DOI:** 10.1186/1471-2407-8-324

**Published:** 2008-11-07

**Authors:** Tai-An Chiang, Ping-Ho Chen, Pei-Fen Wu, Tsu-Nai Wang, Po-Ya Chang, Albert Min-Shan Ko, Ming-Shyan Huang, Ying-Chin Ko

**Affiliations:** 1 Department of Medical Technology, College of Medicine and Life Science, Chung-Hwa University of Medical Technology, Tainan, Taiwan; 2 Division of Environmental Health and Occupational Medicine, National Health Research Institutes, Taiwan; 3 Department of Occupational Safety and Hygiene, Tajen University, Pingtung, Taiwan; 4 Faculty of Public Health, Kaohsiung Medical University, Kaohsiung, Taiwan; 5 Department of Healthcare Administration, Tajen University, Pingtung, Taiwan; 6 Center of Excellence for Environmental Medicine, Kaohsiung Medical University, Kaohsiung, Taiwan; 7 Division of Chest Medicine, Department of Internal Medicine, Kaohsiung Medical University, Kaohsiung, Taiwan; 8 Department of Public Health, Faculty of Medicine, College of Medicine, Kaohsiung Medical University, Kaohsiung, Taiwan

## Abstract

**Background:**

This study used a large-scale cancer database in determination of prognostic factors for the survival of lung cancer subjects in Taiwan.

**Methods:**

Total of 24,910 subjects diagnosed with lung cancer was analysed. Survival estimates by Kaplan-Meier methods. Cox proportional-hazards model estimated the death risk (hazard ratio (HR)) for various prognostic factors.

**Results:**

The prognostic indicators associated with a higher risk of lung cancer deaths are male gender (males versus females; HR = 1.07, 95% confidence intervals (CI): 1.03–1.11), males diagnosed in later periods (shown in 1991–1994 versus 1987–1990; HR = 1.13), older age at diagnosis, large cell carcinoma (LCC)/small cell carcinoma (SCC), and supportive care therapy over chemotherapy. The overall 5-year survival rate for lung cancer death was significantly poorer for males (21.3%) than females (23.6%). Subjects with squamous cell carcinoma (SQCC) and treatment by surgical resection alone had better prognosis. We find surgical resections to markedly increase 5-year survival rate from LCC, decreased risk of death from LCC, and no improved survival from SCC.

**Conclusion:**

Gender and clinical characteristics (i.e. diagnostic period, diagnostic age, histological type and treatment modality) play important roles in determining lung cancer survival.

## Background

Lung cancer is the leading cause of cancer deaths worldwide with geographic and demographic distinctions. Age-standardized incidence rate adjusted by world population (ASRW) for lung cancer is 35.5 per 100,000 males and 12.1 per 100,000 females in 2002, in the world [1]. The highest ASRW for males is in central-Eastern Europe and Northern America with 60 per 100,000, for females is in Northern America with 35.6 per 100,000, and most differences between genders in central-Eastern Europe (M: F = 7.55: 1). Lung cancer is a highly malignant neoplasm with poor prognosis when diagnosed at an advanced stage, and prognostication is crucial for clinicians. Many factors may influence lung cancer survival, including gender [2,3], diagnostic age [4,5], histological type [6,7], and treatment modality [8-10].

For the Taiwanese in 2002, age-adjusted incidence rate of lung cancer was 38.38 per 100,000 males and 19.62 per 100,000 females (M: F = 1.96:1), ranked the second most prevalent cancer in males and fifth in females. Similarly, the age-adjusted mortality rate was 37.75 and 17.85 per 100,000 for males and females, respectively (M: F = 2.11: 1) [11]. Gender has not yet been analyzed as a predictor of survival rate, despite lung cancer incidence and mortality rates showing a male predominance and prognostic factors have been controversial. Present study determines, by gender, the prognostic characteristics of lung cancer subjects in Taiwan. We used a large-scale cancer database from Taiwan Cancer Registry (TCR) to disclose prognostic differences in survival between genders as opposed to a short-term analysis on local hospital data.

## Methods

### Study data

Two systems enlisted. The Taiwan Cancer Registry is a large population-based database established by the National Department of Health provided information on lung cancer subjects (ICD-9 code 162). All discharge notes and data of patient's primary diagnosis of cancer were reviewed by registry-trained personnel in every hospital. This practice remained consistent and unaltered up to this date. Cancer cases from hospitals of at least 50 beds have notified and forwarded to National Department of Health on a compulsory basis. Almost every such hospital participated in this schema (total more than 185 hospitals). Cancer was diagnosed both clinically and histopathologically, and data checked for accuracy and completeness at the National Department of Health, only validated data stored. Proportion of lung cancer cases histo-pathologically diagnosed in the TCR database is greater than 80% [11]. The other system, the mortality database, received standardized death certificates, made mandatory to physicians by the National Department of Health. The vital statistics published by the National Health Department is very complete, with a physician confirmation rate of 99%. Our study population (N = 24,910) comprising of the diagnosed lung cancer subjects from 1987 to 1994, recruited via the TCR system and followed-up, matched correspondingly into the mortality database. Subjects' survival days post-diagnosis were ascertained by an active validation of their vital status until December 31, 2000.

### Descriptive prognostic features

Data accrued in this study include gender, resident area, diagnostic age, histological type at diagnosis, course of treatment, date, and cause of death. Subjects were grouped into three ethnicities, according to their area of residence where the community exceeds 80% of the area's population, the aboriginal Taiwanese, the Hakka, and Hokkien [12]. Histological types defined by ICD-O coding system. Lung cancer subjects were grouped into pathological (76%), clinical (10%) and imaging (14%) diagnoses [11]. Pathological diagnosis pertained to several histological subtypes, such as carcinoma not otherwise specified (NOS: M8000-M8004, M8010-M8011, M8032-M8034), squamous cell carcinoma (SQCC: M8050-8076), adenocarcinoma (M8140, 8211, 8230–8231, 8250–8260, 8323, 8550–8560, 8570–8572), small cell carcinoma (SCC: M8040-8045), large cell carcinoma (LCC: M8012-8031, M8310), and other carcinomas [13]. The treatment modalities examined were surgical resection alone, radiation therapy (RT) alone, chemotherapy (CT) alone, supportive care therapy (ST) alone (prescribing morphine for cancer pain, oxygen for symptomatic care of chronic obstructive pulmonary disease (COPD), antidepressants in treatment of depression within hospital care), and other complex therapy (immunotherapy, hormonal therapy, and traditional Chinese herbal medicine therapy), and unknown therapy [11].

### Statistical analysis

Lung cancer death (after eliminated other competing causes of death) was treated as two distinct outcomes for the purpose of this study. In our lung cancer survival analysis, deaths fulfilling the ICD-162 criteria were classified as deaths from lung cancer, whereas those who died from other causes or still alive became censored observations. Using log-rank test, the gender curves differed significantly for lung cancer death, so the subjects were segregated into male and female groups. Frequency distributions on demographic, clinical and therapy characteristics on gender were compared by the chi-square test. Survival days after diagnosis were estimated by Kaplan-Meier and 5-year survival rates (absolute survivals) presented. For each gender, we conducted relative survival analysis (defined as observed survival divided by expected survival, adjusting for expected mortality that the cohort would have from all causes of death [14]). Survival rates differed significantly for each clinical factor outcome by gender with the log-rank test. After log-log survival plots were used to verify the proportion hazard assumptions for each predictor, the Cox proportional-hazards model was applied to estimate their contributions. The adjusted multiple variables of prognostic factors (diagnostic period, diagnostic age, resident area, histological type diagnosis, therapeutic choice) were used in assessing the hazard ratio (HR) of their relationships, based on gender survival. All analyses used SAS version 8.2 statistical software (SAS Institute, Inc., Cary, NC, USA).

## Results

### Distribution of prognostic characteristics by gender (Table 1)

**Table 1:** Characteristics of lung cancer subjects at time of diagnosis by gender in 1987–1994

	**Males** **(N = 18,056)**	**Females** **(N = 6854)**	
			
**Characteristics**	**N (%)**^a^	**N (%)**^a^	***p *Value**
Mean age (years) ± S.D.	64.8 ± 9.9	62.2 ± 14.5	< 0.0001
Period of diagnosis (years)			
1987–1990	7455 (41)	2690 (39)	0.003
1991–1994	10601 (59)	4164 (61)	
Diagnostic age (yrs)			
<= 39	418 (2)	382 (6)	< 0.0001
40–49	855 (5)	730 (11)	
50–59	3069 (17)	1409 (21)	
60–69	7364 (41)	2137 (31)	
>= 70	6320 (35)	2196 (32)	
Resident area			
Aboriginal community	410 (2)	128 (2)	0.0060
Hakka community	1462 (8)	491 (7)	
Hokkien community	16184 (90)	6235 (91)	
Histological type			
SQCC	5855 (32)	1003 (15)	< 0.0001
Adenocarcinoma	4880 (27)	3156 (46)	
SCC	2005 (11)	276 (4)	
LCC	326 (2)	107 (2)	
Carcinoma, NOS	1664 (9)	599 (9)	
Other carcinoma	3326 (18)	1713 (25)	
Treatment modality			
Surgery resection	2360 (13)	859 (13)	< 0.0001
RT	3970 (22)	1078 (16)	
CT	1974 (11)	597 (9)	
ST	4316 (24)	2085 (30)	
Other complex therapy	40 (< 1)	23 (< 1)	
Unknown therapy	5396 (30)	2212 (32)	

*Abbreviations: *S. D., standard deviation.

^a ^May not total 100% due to rounding.

SQCC: squamous cell carcinoma; SCC: small cell carcinoma; LCC: large cell carcinoma.

NOS: not otherwise specified.

RT: radiation therapy; CT: chemotherapy; ST: supportive care therapy.

Total of 24,910 subjects (72% males and 28% females) were analysed during the period 1987–1994. Results showed that each characteristic to be strongly associated with gender. Males were diagnosed at a significantly older age than females. Particularly, in both genders, the proportion of subjects was significantly higher in 1991–1994 than 1987–1990. For the proportion of diagnostic age, females were predominantly higher than males in younger age groups (<= 59 years). Males were significantly more likely to present with SQCC than other types, whereas females were significantly more likely to present with adenocarcinoma than other types. With regards to the therapeutic choice, except for unknown therapy, ST was the most frequented treatment modality, followed by RT, surgical resection, and CT in both genders. Females were more likely to choose ST and unknown therapy versus a significantly higher proportion of males treated with RT and CT therapy.

### Survival of males and females associated with death from lung cancer (Table 2)

**Table 2:** Association between the prognostic characteristics and 5-year survival rate in lung cancer subjects

**Characteristics**	**Lung cancer death (%)**		
	
	**Males** **(N = 12658)**	**Females** **(N = 4571)**	***p *Value**^a^
All subjects	21.3	23.6	< 0.0001
Period of diagnosis (years)			
1987–1990	23.6	24.1	0.3917
1991–1994	19.7	23.2	< 0.0001
***p *Value**^a^	< 0.0001	0.6865	
Diagnostic age (years)			
<= 39	30.9	34.5	0.5380
40–49	27.0	25.0	0.7519
50–59	22.8	22.4	0.2629
60–69	21.3	22.9	0.1300
>= 70	20.1	23.3	0.0011
***p *Value**^a^	< 0.0001	< 0.0001	
Residence area			
Aboriginal community	29.6	17.3	0.1060
Hakka community	21.7	21.8	0.5235
Hokkien community	21.5	23.8	< 0.001
***p *Value**^a^	0.0578	0.2972	
Histological types			
SQCC	21.5	22.5	0.9190
Adenocarcinoma	18.8	19.5	0.0073
SCC	14.2	15.9	0.1058
LCC	17.8	18.0	0.7648
Carcinoma, NOS	18.2	18.8	0.0534
Other carcinoma	34.2	38.1	0.0012
***p *Value**^a^	< 0.0001	< 0.0001	
Treatment modality			
Surgery resection	37.4	42.3	0.0001
RT	15.3	14.5	0.4353
CT	15.3	15.8	0.2509
ST	23.3	24.7	0.0094
Other complex therapy	18.4	13.7	0.5387
Unknown therapy	20.2	22.3	0.0087
***p *Value**^a^	< 0.0001	< 0.0001	

SQCC: squamous cell carcinoma; SCC: small cell carcinoma; LCC: large cell carcinoma. NOS: not otherwise specified. RT: radiation therapy; CT: chemotherapy; ST: supportive-care therapy. ^a ^Log-rank test

**Figure 1:**
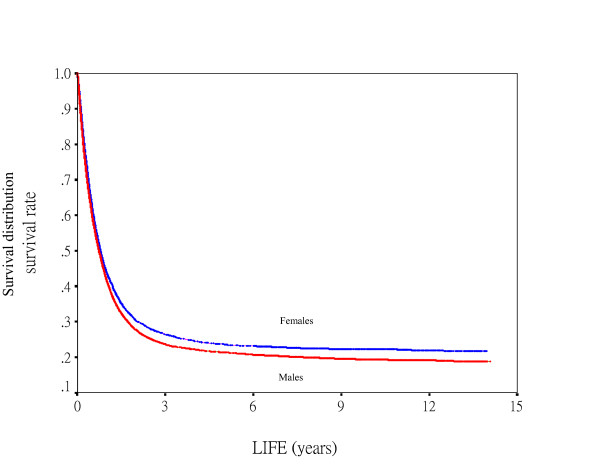
Survival curves for gender according to lung cancer death (N = 17229, *p *< 0.0001) as the endpoints.

During our follow-up period, total of 17,229 (69%) subjects died from lung cancer. The observed 5-year rate of survival for lung cancer death was 21.9% (21.3% for males, 23.6% for females), and 5-year relative survival was 32.4% (31.4% for males, 35.1% for females). Median survival was 204.5 days for males and 213.5 days for females. Fig. 1 denotes males having significantly lower survival curve than females.

For males, a significantly reduced survival rate was observed in 1991–1994 than 1987–1990. And in the period 1991–1994, the 5-year survival rates of females significantly higher than males. In terms of both genders, the 5-year survival rates were significantly higher in the younger diagnostic age groups than older groups. For histological diagnosis, males had a significantly lower survival estimate for adenocarcinoma than females, and subjects with SCC have the lowest survival rates (14.2% for males and 15.9% for females). When comparing treatment modalities, the highest 5-year survival rates were 37.4% in males and 42.3% in females, for surgical resection alone. Surgery alone, ST alone and unknown therapy showed females had significantly higher survival rates than males.

### Contribution to prognostic characteristics for lung cancer death by each gender (Table 3)

**Table 3:** Multivariate proportional hazard ratio for lung cancer death by gender

**Characteristics**	**Lung cancer death (N = 17,229)**	
	
	**Males**	**Females**
	
	**HR (95% CI)**	**HR (95% CI)**
Period of diagnosis (years)		
1987–1990	1.00 -	1.00 -
1991–1994	1.13 (1.09–1.18)*	1.03 (0.97–1.10)
Diagnostic age (years)		
<= 39	1.00 -	1.00 -
40–49	1.07 (0.93–1.25)	1.17 (1.00–1.37)
50–59	1.25 (1.10–1.42)*	1.24 (1.07–1.43)*
60–69	1.31 (1.15–1.48)*	1.30 (1.13–1.50)*
>= 70	1.43 (1.26–1.62)*	1.37 (1.19–1.58)*
Resident area		
Hokkien community	1.00 -	1.00 -
Aboriginal community	0.85 (0.75–0.96)*	1.13 (0.92–1.40)
Hakka community	1.00 (0.94–1.07)	1.02 (0.91–1.14)
Histological type		
SQCC	1.00 -	1.00 -
Adenocarcinoma	1.16 (1.11–1.21)*	1.06 (0.98–1.16)
SCC	1.25 (1.17–1.33)*	1.07 (0.91–1.25)
LCC	1.38 (1.21–1.57)*	1.24 (0.98–1.56)
Carcinoma, NOS	1.16 (1.09–1.24)*	1.02 (0.90–1.15)
Other carcinoma	0.78 (0.73–0.82)*	0.66 (0.60–0.73)*
Treatment modality		
CT	1.00 -	1.00 -
Surgery resection	0.60 (0.56–0.65)*	0.49 (0.43–0.56)*
RT	1.11 (1.04–1.19)*	1.07 (0.95–1.20)
ST	1.17 (1.10–1.26)*	1.10 (0.98–1.23)
Other complex therapy	1.43 (1.00–2.05)	1.20 (0.75–1.93)
Unknown therapy	1.11 (1.04–1.18)*	1.02 (0.92–1.14)

SQCC: squamous cell carcinoma; SCC: small cell carcinoma; LCC: large cell carcinoma. NOS: not otherwise specified. RT: radiation therapy; CT: chemotherapy; ST: supportive care therapy. HR: hazard ratio; CI: confidence interval. * Statistical significance (*p *< 0.05).

Diagnostic period was a significant predictor of lung cancer mortality in males, most evident in the declining survival trend in 1991–1994. For subjects aged 50–59 years, 60–69 years and over 70 years, they were at significantly higher risk of dying than those aged 39 years or younger. For the residence area, males from the Taiwanese aboriginal community have significantly lowest risk of mortality (HR= 0.85) than the Hokkien community. For the histological types, males with LCC (HR = 1.38) have significantly highest risk of death, than SQCC, followed by SCC (HR = 1.25), adenocarcinoma (HR = 1.16), and carcinoma-NOS (HR = 1.16). The risk of death was significantly lower for other carcinoma (HR = 0.78 and 0.66 for males and females, respectively) than for SQCC. Therapeutic modalities were also significant predictors of survival for both genders. There was a significant improvement in prognosis for subjects treated by surgical resection alone (HR = 0.60 for males and 0.49 for females) than CT alone. But males who received ST, RT and unknown therapy have a significant deleterious effect (HR = 1.17, 1.11 and 1.11, respectively).

### Effects of the covariables on hazard ratio for lung cancer death by gender (Table 4)

**Table 4:** Crude and adjusted hazard ratio for males versus females according to lung cancer death

	**Lung cancer death** **(N = 17,229)** **M: F**^a^**HR (95% CI)**
**Crude hazard ratios**	1.09 (1.05–1.12)*
**Adjusted hazard ratios**	
Adjusted for the period of diagnosis (years)	1.09 (1.05–1.13)*
Above plus diagnostic age, resident area	1.06 (1.03–1.10)*
Above plus morphological type, treatment modality	1.07 (1.03–1.11)*

*Abbreviations: *HR: hazard ratio; CI = confidence interval.

^a^M:F: male -to- female ratio.

* Statistical significance (*p *< 0.0001).

The crude hazard ratio for males was 1.09 (95% CI: 1.05–1.12) for lung cancer death. After adjusted for all prognostic factors, males have significantly higher risk of death than females (HR = 1.07).

## Discussion

The five-year survival rate of lung cancer patients in Taiwan was poorer than other cancers [15]. European Union study [16] and United States study [17] have similarly shown no improvements in prognosis for lung cancer patients in the two decades prior death. Our Kaplan-Meier analysis showed that 5-year survival rate of lung cancer death was higher than in Western population, possibly because the 5-year survival rate of lung cancer death has eliminated other competing causes of death [3,8,17]. Past studies have focused on differences in incidence and histological types of lung cancer by gender [16,18,19]. Present study showed survival and prognostic factors of lung cancer by gender from lung cancer databases, and lung cancer death as outcome used in effort to clear arbitrary effect of death from other causes.

### Gender differences in the survival of subjects diagnosed with lung cancer

Our findings showed the long-term survival rate was significantly better for females, and the average diagnostic age was significantly higher for males, as consistent with other publications on gender and survival of lung cancer patients [2,3,7,20]. Our large lung cancer database confirmed gender to be an independent predictor of lung cancer prognosis by the multivariate Cox analysis, moreover males diagnosed with lung cancer had a risk 1.07 times higher than females.

### Prognostic factors in the survival of subjects diagnosed with lung cancer

The proportion of lung cancer subjects in 1991–1994 was significantly higher than 1987–1990, during which lung cancer posed diagnostic and therapeutic challenges, with extremely poor prognosis. Our finding showed, for males, the mortality to be significantly higher and survival rates to be significantly lower in the periods 1991–1994 than 1987–1990. Possibly, the declining survival rates reflected no improvements to early cancer detection or treatment efficacy, and warrants further attention.

In review of diagnostic age on lung cancer death, increasing age, especially older than 70 years, remained significant risk factor in both genders. Other reports also noted increasing age possessed an adverse effect on survival for lung cancer patients [4,5,16,21]. There may be poorer tolerance to treatment, and health conditions in older patients. Differing opinions exist regarding relationship between diagnostic age and survival rate in lung cancer patients [22,23].

For histological distribution, SQCC has frequently occurred in males and adenocarcinoma in females. The lowest 5-year survival rate was seen in both genders with SCC. Similar evidence has been shown by studies conducted in Norway [3] and France [24]. After adjusted for diagnostic period, diagnostic age, resident area, and treatment modality, males with LCC had a significantly highest risk in terms of mortality. The impact of histological diagnosis was notable in males with adenocarcinoma, SCC and LCC, have significantly poorer prognosis compared to SQCC type. Males also have significantly lower survival estimate of adenocarcinoma than females. A switch in occurrence of SQCC in Taiwanese men to adenocarcinoma, SCC and LCC was observed in 1987 to 2003 [11]. These results could partially explain for the poor prognosis in males.

Currently, complete surgical resection offer effective therapy for lung cancer patients [6,10]. This study showed surgical resection to markedly improve outcome for both genders, and survival by surgical resection was significantly better for females. Previous studies also noted females to have better postoperative survival and prognosis [2,3,25,26]. Differences in aspects of medical intervention may have accounted for gender disparity in terms of survival rates. We showed surgical resection to be protective. As in another study, surgical resection was also beneficial to survival [4,6,10,16].

Subjects treated with ST alone or did not receive any specific treatment for lung cancer have unfavorable prognosis [4,9,16]. In this study, 5-year survival rate of ST alone was better than RT, CT in both genders. But after adjusted for other prognostic factors, 5-year survival rate of ST therapy have markedly deleterious effect. Except for unknown therapy, females were more likely to choose ST therapy than males. From clinical expertise, physicians specialized in chest medicine considered this possibly due to females fearing the adverse effects (such as lose hair, vomit, etc) of RT, CT therapy. Females spent more time accessing the health care system, the disease was therefore found at an earlier diagnostic age than males (17% for females and 7% for males are under 50 years of age). Johnston *et al*. showed hormonal role may also influence survival rates in terms of gender where androgens may have stimulatory effect on lung cancer growth [20].

Lung cancer survival has strongly dependent upon the staging [2,3,6,16]. This was a limitation in this study, as data pertained to exact staging was unavailable on the TCR. Zhang *et al*. has recommended surgery for stage I, II and stage IIIa in non-SCC and stage I, II in SCC [6]. Data on early clinical staging of cancer could be available for surgical resection alone. But treatment by CT alone, RT, ST alone meant diagnoses were made at advanced stages of the disease. Several researchers obtained similar results [2,16]. As result, we evaluated surgical effects on survival instead of clinical stages. In our analytical procedure, subjects were categorized into surgery and non-surgery therapy, and survival rates of these two groups compared. Results indicated the survival of surgery group to be significantly higher than the non-surgery group (data not shown). Except for SCC patients, the 5-year survival of histological types showed significant differences between patients with or without surgery (Table 5). Surgical resection has markedly increased 5-year survival, decreased the risk of death of LCC patients, but no improvement to the survival of SCC patients. As staging is unavailable to TCR, individual subjects' therapeutic choices could be treated as a clinical reference.

**Table 5:** Five-year survival rates and multivariate proportional hazard ratio of lung cancer death by surgical status

**Characteristics**	**Lung cancer death** **(N = 17,229)**		
	**Surgery**	**Non-surgery**	

	**Survival rates (%)**	**Survival rates (%)**	***p *Value**^a^
	**HR (95% CI)**	**HR (95% CI)**	

All subjects	38.8	19.3	< 0.0001
Histological types			
SQCC	41.6	16.9	< 0.0001
	1.00 -	1.00 -	
Adenocarcinoma	36.5	14.7	< 0.0001
	1.14 (1.03–1.26)*	1.14 (1.09–1.19)*	
SCC	17.1	14.3	0.5716
	2.49 (2.05–3.03)*	1.13 (1.07–1.20)*	
LCC	43.7	11.9	< 0.0001
	1.01 (0.74–1.38)	1.42 (1.26–1.61)*	
Carcinoma, NOS	31.7	17.5	0.0001
	1.53 (1.24–1.89)*	1.12 (1.05–1.18)*	
Other carcinoma	65.0	34.1	< 0.0001
	0.59 (0.45–0.78)*	0.77 (0.74–0.81)*	

SQCC: squamous cell carcinoma; SCC: small cell carcinoma; LCC: large cell carcinoma.

NOS: not otherwise specified.

RT: radiation therapy; CT: chemotherapy; ST: supportive care therapy. ^a^Log-rank test. HR: hazard ratio; CI: confidence interval. * Statistical significance (*p *< 0.05).

In addition to the factors aforementioned, cigarette smoking has been well-known risk factor for lung cancer patients to dramatically decrease survival rates [5,8,24,27]. But information on cigarette smoking was unavailable on the TCR until 2007 in Taiwan. The causal factors of lung cancer between males and females have been heterogeneous [18]. Statistics showed 86% of male lung cancer and 9–10% of female lung cancer as having social history of cigarette smoking [28], could explain the poorer prognosis in Taiwanese males. Cooking oil fume and environmental tobacco smoke accounted for the largest proportion of attributable risk of lung cancer in women [18,28]. Taiwanese women have relatively high lung cancer incidence in spite very few smokers. However, smoking behavior could not fully explain the differences in epidemiological characteristics of lung cancer between males and females in Taiwan. Many reports from North America have similar smoking rates in gender, still found females to have better survival than males [17,20,29]. Gender is an independent factor to dominate the survival of lung cancer.

## Conclusion

This study analysed the long-term survival of lung cancer patients from the population data in the TCR, and identified gender as an important and independent prognostic factor for lung cancer death patients. Clinical characteristics (i.e. diagnostic period, diagnostic age, histological type, treatment modality) have important roles in lung cancer survival.

## Competing interests

The authors declare that they have no competing interests.

## Authors' contributions

TA and PH carried out the study, participated in the sequence alignment and drafted the manuscript. PF, and TN carried out the data compilation and drafted the manuscript. PY, and Albert participated in the design of the study and performed the statistical analysis. MS participated in the sequence alignment. YC conceived of the study, and participated in its design and coordination. All authors read and approved the final manuscript.

## Pre-publication history

The pre-publication history for this paper can be accessed here:


